# Signal Quality Improvement Algorithms for MEMS Gyroscope-Based Human Motion Analysis Systems: A Systematic Review

**DOI:** 10.3390/s18041123

**Published:** 2018-04-06

**Authors:** Jiaying Du, Christer Gerdtman, Maria Lindén

**Affiliations:** 1 School of Innovation, Design and Engineering, Mälardalen University, 721 23 Västerås, Sweden; maria.linden@mdh.se; 2 Motion Control i Västerås AB, 721 30 Västerås, Sweden; christer.gerdtman@motioncontrol.se

**Keywords:** drift, MEMS gyroscope, motion analysis, motion sensors, noise/error reduction, signal processing algorithms, systematic review

## Abstract

Motion sensors such as MEMS gyroscopes and accelerometers are characterized by a small size, light weight, high sensitivity, and low cost. They are used in an increasing number of applications. However, they are easily influenced by environmental effects such as temperature change, shock, and vibration. Thus, signal processing is essential for minimizing errors and improving signal quality and system stability. The aim of this work is to investigate and present a systematic review of different signal error reduction algorithms that are used for MEMS gyroscope-based motion analysis systems for human motion analysis or have the potential to be used in this area. A systematic search was performed with the search engines/databases of the ACM Digital Library, IEEE Xplore, PubMed, and Scopus. Sixteen papers that focus on MEMS gyroscope-related signal processing and were published in journals or conference proceedings in the past 10 years were found and fully reviewed. Seventeen algorithms were categorized into four main groups: Kalman-filter-based algorithms, adaptive-based algorithms, simple filter algorithms, and compensation-based algorithms. The algorithms were analyzed and presented along with their characteristics such as advantages, disadvantages, and time limitations. A user guide to the most suitable signal processing algorithms within this area is presented.

## 1. Introduction

In contemporary/modern society, demographic changes of population and multiple diseases lead to increasing demands on and costs for the healthcare systems [1]. Thus, home-based wearable self-controlled medical sensor systems have become a research topic of interest in the healthcare area, in which human motion analysis is important because of its crucial applications. For example, sensor systems for rehabilitation, athletic performance evaluation/analysis, and monitoring of health for the elderly who are alone at home are in extremely high demand [2].

Microelectromechanical system (MEMS) sensors have been widely used in many areas where miniature sensors that are of a low cost and low weight are desired [3]. MEMS technology has enabled the development of miniaturized inertial sensors, which have been used in motor activity and other health status monitoring systems [4]. They have already been widely applied in motion analysis systems in the medical field for knee/ankle joint measurement [5,6,7,8], gait analysis [9,10], ambulatory measurement and analysis of the lower limbs [11,12], the collection of anatomical joint angles during stair ascent [13], hand gesture recognition [14,15,16], head-motion-controlled wheelchairs [17], a head-motion-controlled mouse [18,19], and digital motion analysis systems for rehabilitation from impairments such as those caused by accidents or stroke [20,21]. An MEMS-based small wearable embedded sensor system for motion analysis is a typical solution for a free-living measurement environment.

MEMS-based motion sensors such as MEMS gyroscopes and MEMS accelerometers are microscale inertial sensors that have the advantages of a small size, light weight, low cost, low power consumption, high sensitivity, and high precision [22]. However, the resolution and stability of MEMS inertial sensors are not adequate and must be improved in sensor networking, for example, for the monitoring of human motion, the distribution of earthquakes, and the vibration of buildings [23]. MEMS gyroscope signal errors are often due to their high sensitivity to environmental disturbances such as shocks, vibrations, and temperature changes [24]. For human motion analysis with wearable sensor systems, large amounts of noise, such as human tremors and environmental vibrations, are included in the measurement signals [25,26,27]. For position measurements, the angular position can be determined by integrating the measured MEMS gyroscope signal. One step of integration is needed from the measured MEMS gyroscope signal to the angular position. However, errors are accumulated by the integration. Accumulated error can also be regarded as drift due to numerical integration during the position calculation [28]. Signal processing algorithms/methods are an important part of motion analysis system development with MEMS gyroscopes, which should be able to minimize the signal errors, improve the signal quality, and further improve the system stability.

The aim of this paper is to present a systematic review of the signal error reduction algorithms/methods that are used for MEMS gyroscope-based motion analysis systems for human motion analysis or have the potential to be used in this area. The content is presented as a user guide for selecting the most suitable signal processing algorithms for MEMS gyroscopes in various situations. 

## 2. Methodology/Methods

### 2.1. Inclusion and Exclusion Criteria

The inclusion criteria were as follows:

1. The article was published as a journal article or a conference paper in English.
2. The article was published in the past 10 years (between 2007 and 2017).
3. The primary subject of the study was signal error reduction methods/algorithms for MEMS gyroscope-based motion analysis systems that are intended for human motion analysis or have the potential to be used in this area.

Articles were excluded from this paper if they instead focused on avoiding errors in the development and fabrication of the MEMS gyroscope inertial structures or on systems that were not intended for the application of human motion analysis but for other applications, e.g., navigation, global positioning, attitude compensation, space contrail detection, missile control, and trajectory analysis. Articles were also excluded if they focused on methods that were not intended for signal error reduction or signal quality improvement, but rather for other special areas such as fall detection, pedestrian indoor localization, and motion/gesture recognition.

### 2.2. Searching Strategy and Analysis

With the inclusion and exclusion criteria specified above, the search was performed in the following electronic databases: Association for Computing Machinery (ACM) digital library within the area of computer science [29]; IEEE Xplore with the contents of engineering science, scholarly journals, conference reports, and IEEE standards [30]; PubMed with the contents of medicine and health science and scholarly journal articles [31]; and Scopus, which is a multidisciplinary citation database [32]. The searched keyword string was set as (((signal processing OR noise OR drift OR error OR reduction) AND motion*) AND “MEMS gyroscope”). 

Through database searching without a time limitation, 33 results from 2001 to 2017 were initially found in ACM; 120 results from 1999 to 2017 were found in IEEE Xplore; three results from 2010 to 2016 were found in PubMed; and 35 results from 2005 to 2017 were found in Scopus. Within the publishing year limitation of the most recent 10 years, namely, from 2007 to 2017, 165 (28 from ACM + 104 from IEEE + three from PubMed + 30 from Scopus) results remained. After eliminating the duplicates, 148 results remained. The main author read through the titles and abstracts of the retrieved results and performed an initial analysis to determine whether the inclusion criteria were fulfilled or not. The full texts of those articles that fulfilled the inclusion criteria based on the title and abstract were assessed in detail by the main author. Irrelevant articles were excluded based on the exclusion criteria. The eligibility was checked.

**Figure 1:**
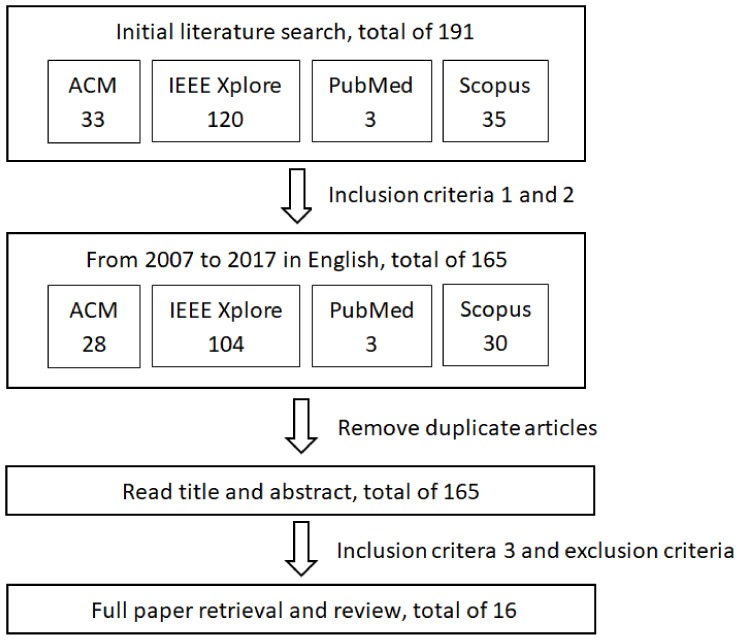
Research method: Flow diagram of the search and review procedure.

The searching and review procedure is illustrated in Figure 1.

## 3. Results

**Figure 2:**
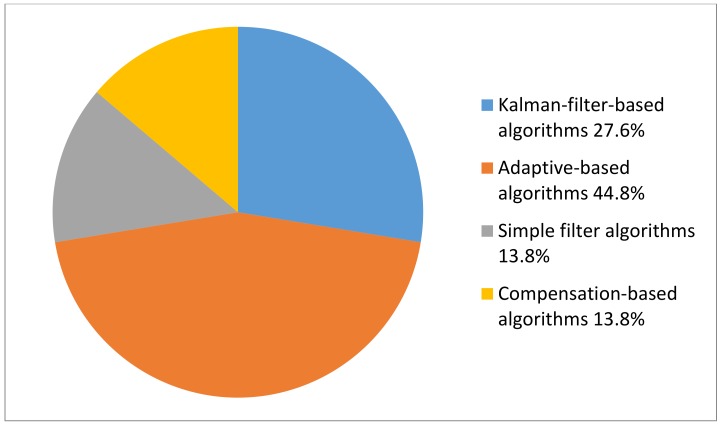
Proportions of the four types of algorithms in the reviewed results. (Kalman-filter-based algorithms 27.6%; Adaptive-based algorithms 44.8%; Simple filter algorithms 13.8%; Compensation-based algorithms 13.8%).

From the 16 reviewed articles, 17 algorithms/methods were identified. They were categorized into four groups, namely, Kalman-filter-based algorithms, adaptive-based algorithms, simple filter algorithms, and compensation-based algorithms, and are presented below. All algorithms/methods were aimed at reducing different signal errors for the MEMS gyroscope-based human motion analysis system (or had the potential for human applications). The proportions of the four types of algorithms in the reviewed results are shown in Figure 2.

### 3.1. Kalman Filter (KF)-Based Algorithms

#### 3.1.1. Kalman Filter

The Kalman filter is a common filter that is used for sensors. It consists of a loop that contains two steps: time updating, which is a prediction process, and measurement updating, which is a correction process [33]. The Kalman filter process is as follows:

- First, initial estimates for ${\hat{x}}_{k-1}$ and $P_{k-1}$ are obtained.
- Then, the two-step loop is entered, as shown below:

Time updating process:

(1) $${\hat{x}}_{k}^{-}=A{\hat{x}}_{k-1}+Bu_{k-1}$$

(2) $$P_{k}^{-}=AP_{k-1}A^{T}+Q$$

Measurement updating process:

(3) $$K_{k}=P_{k}^{-}H^{T}{\left(HP_{k}^{-}H^{T}+R\right)}^{-1}=\frac{P_{k}^{-}H^{T}}{HP_{k}^{-}H^{T}+R}$$

(4) $${\hat{x}}_{k}={\hat{x}}_{k}^{-}+K_{k}(z_{k}-H{\hat{x}}_{k}^{-})$$

(5) $$P_{k}=(I-K_{k}H)P_{k}^{-}$$

where $\hat{x}$ is the posteriori estimated state, ${\hat{x}}^{-}$ is the priori state, u is the control vector, z is the measurement signal, k is a discrete point in time, A is the state transition model, B is the control input model, P is the error covariance, Q is the process noise covariance, K is the Kalman gain, H is the measurement matrix, R is the measured noise covariance, and I is the identity matrix. 

Some common applications of the Kalman filter are noise reduction and signal prediction and estimation. It is one of the most common algorithms for sensor problems such as gyro sensor drift compensation [34].

Kalman filters have also been applied for human tremor estimation [35,36] and can improve the human-operated MEMS gyroscope signal by removing human tremors.

#### 3.1.2. Discrete KF in an Optimal Approach

**Figure 3:**
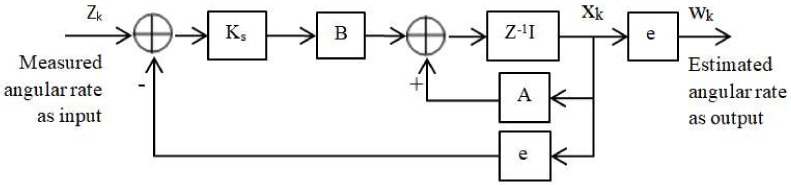
Estimation process of the discrete Kalman filter.

A Kalman filter is designed based on a steady-state filter gain that is obtained from an analysis of Kalman filter observability with the aim of reducing the bias drift and noise from the outputs signal of the MEMS gyroscope [37]. The Kalman filter is designed with a system state vector that is modeled based on both the true angular rate ω and the bias drift b. The parameters of covariance matrices Q and R are derived from the noise variance of the angular random walk (ARW) and the rate random walk (RRW) and the variance $q_{\omega }$. The steady-state Kalman filter gain $K_{s}$ is analyzed off-line in advance. Parameters *A* and *B* are calculated based on the eigenvector matrix *S* and eigenvalues $\lambda_{1}$ and $\lambda_{2}$. Finally, with these parameters, the discrete-time KF is applied as shown in Figure 3, which is derived from the figure in the original paper.

#### 3.1.3. Simplified Basic KALMAN Filter

**Figure 4:**
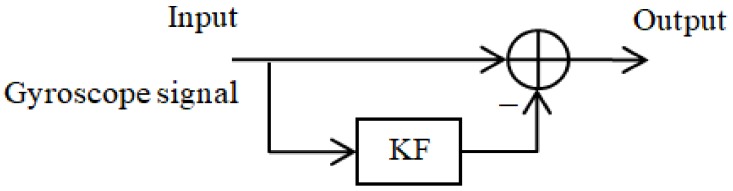
Structure of the Kalman filter algorithm for drift/offset estimation.

The simplified basic Kalman filter can be used to reduce the noise [25] and temperature drift [26,27]. It is used digitally/discretely to reduce the noise and to estimate the temperature drift trend. The Kalman filter is used to estimate the offset and drift trend. The structure of drift/offset reduction by the Kalman filter is illustrated in Figure 4.

#### 3.1.4. Kalman-Filter-Based Position Estimation Algorithm

**Figure 5:**
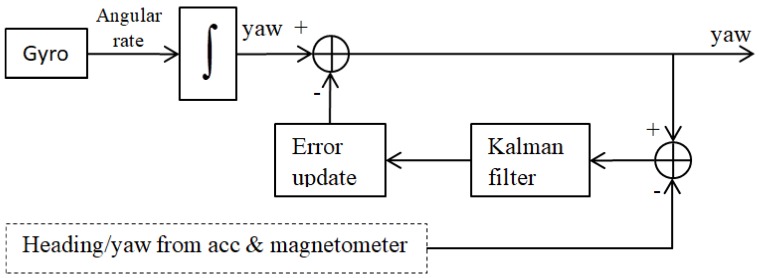
Structure of the Kalman-filter-based position estimation algorithm.

A Kalman-filter-based position estimation algorithm for correcting the yaw was presented by Pedro Neto et al. [38] and is illustrated in Figure 5. Figure 5 is derived from the figure in the original paper and shows the relationship between the MEMS gyroscope, the Kalman filter, and the additional accelerometer and magnetometer.

### 3.2. Adaptive-Based Algorithms

#### 3.2.1. Least Mean Square (LMS) Algorithm

**Figure 6:**
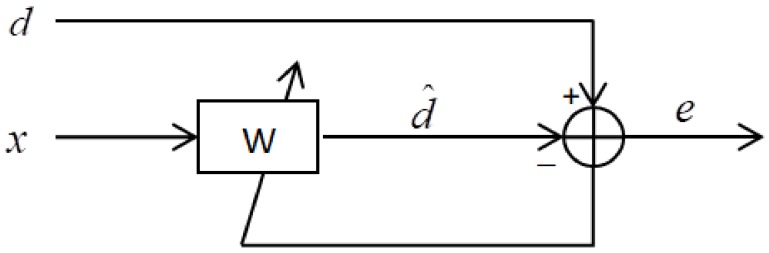
Structure of the LMS adaptive filter.

The LMS algorithm was applied in a MEMS gyroscope-based computer head-borne mouse to reduce the noise [25]. The structure of the LMS adaptive filter is illustrated in Figure 6, where *d* is the desired signal, *x* is the input, $\hat{d}$ is the output of the adaptive filter, *e* is the error signal, and W is the LMS adaptive filter.

#### 3.2.2. Adaptive Sliding Mode Controller

**Figure 7:**
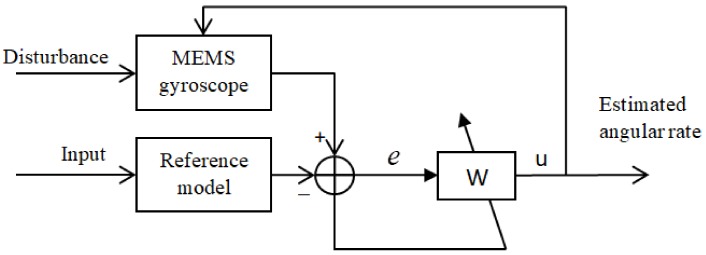
Indirect adaptive sliding mode control for an MEMS gyroscope. (W is the indirect sliding mode controller with an adaptive law).

The adaptive sliding mode controller for the MEMS gyroscope was introduced in [39], which can compensate in real time for the fabrication imperfections and estimate the angular velocity and the damping and stiffness coefficients. The block diagram is shown in Figure 7, where W stands for the adaptive indirect sliding mode controller. Figure 7 is derived from Figure 2 in the original paper.

#### 3.2.3. Adaptive Bandpass Filter (ABPF)

**Figure 8:**
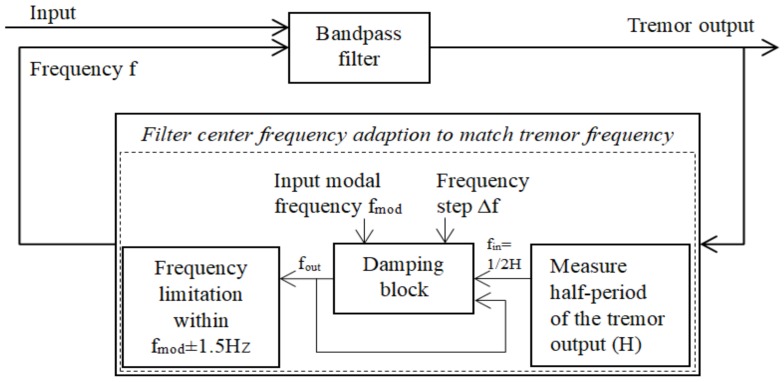
Main structure of the adaptive bandpass filter.

The ABPF algorithm is a basic algorithm for reducing the typical noises and tremors in inertial sensor data [40]. As described in the paper, tremor patient data were collected with both a gyroscope and an accelerometer. A Butterworth second-order bandpass filter with an adapted center frequency was designed in the program in a MATLAB Simulink environment. As shown in Figure 8, the method can be mainly summarized as bandpass filtering and filter center frequency adaption. The bandpass filter transfer function is:

(6) $$H(s)=\frac{\sqrt{2}\omega_{a}s}{s^{2}+\sqrt{2}\omega_{a}s+\omega_{a}^{2}}$$

where $\omega_{a}$ is the filter center frequency, which is adapted in the closed loop based on the dominant frequency of the input signal. In Figure 8, frequency f is equal to 2π$\omega_{a}$. The damping block limits the changes of the tremor frequency estimation with the frequency step ∆f according to the input modal frequency f_mod_ to adjust the speed of the adaptation. 

#### 3.2.4. Weighted-Frequency Fourier Linear Combiner (WFLC) Algorithm

The WFLC algorithm is the most widely used algorithm for tremor modeling [35]. It is a type of adaptive algorithm. It was used to reduce the noise that is associated with human tremors and electrical noise in the application of an MEMS gyroscope-based computer head-borne mouse [25,27]. The discrete WFLC algorithm is:

(7) $$x_{r_{k}}=\{\begin{array}{c} \sin(r\sum_{t=0}^{k}w_{0_{k}}),1\leq r\leq M\\ \cos((r-M)\sum_{t=0}^{k}w_{0_{k}}),M+1\leq r\leq 2M \end{array}$$

(8) $$y_{k+1}=y_{k}+W^{T}X$$

(9) $$\varepsilon_{k}=s_{k}-y_{k}-w_{bias_{k}}$$

(10) $$w_{bias_{k+1}}=w_{bias_{k}}+2\mu_{b}\varepsilon_{k}$$

(11) $$w_{0_{k+1}}=w_{0_{k}}+2\mu_{0}\varepsilon_{k}\sum_{i=1}^{M}\left(w_{i}x_{M+i}-w_{M+i}x_{i}\right)$$

(12) $$W_{k+1}=W_{k}+2\mu_{1}X_{k}\varepsilon_{k}$$

where M is the number of harmonics; $\mu_{0}$, $\mu_{1}$, and $u_{b}$ are the adaptive parameters; $s_{k}$ is the input and $\varepsilon_{k}$ is the error signal at time point *k*; and $W_{k}={\left[w_{1_{k}},...,w_{2M_{k}}\right]}^{T},X_{k}={\left[x_{1_{k}},...,x_{2M_{k}}\right]}^{T}$.

#### 3.2.5. Bandlimited Multiple Fourier Linear Combiner (BMFLC) Algorithm

The BMFLC algorithm is derived from the Fourier Linear Combiner (FLC) and was proposed more recently [35]. Similar to the WFLC algorithm, it can be used to estimate tremors [35]. As shown in the paper [35], the discrete BMFLC algorithm can be described as:

(13) $$x_{r_{k}}=\{\begin{array}{c} \sin(\omega_{0}+(\omega_{f}-\omega_{0})\frac{r-1}{G+1}k),1\leq r\leq M\\ \cos(\omega_{0}+(\omega_{f}-\omega_{0})\frac{r-1}{G+1}k),M+1\leq r\leq 2M \end{array}$$

(14) $$\varepsilon_{k}=s_{k}-W_{k}^{T}X_{k}-\mu_{b}$$

(15) $$W_{k+1}=W_{k}+2\mu X_{k}\varepsilon_{k}$$

where *M* is the number of harmonics; *G* is the number of FLC-filters in between; μ is the amplitude adaptation gain; $\omega_{0}$ and $\omega_{f}$ are the lower and upper frequencies of the FLC bank, respectively; and $\mu_{b}$ is a bias weight.

#### 3.2.6. Sensor Fusion

Sensor fusion approaches are adaptive algorithms that combine sensory data from independent sources, irrespective of their advantages and disadvantages, to optimize the system performance, as mentioned in [41]. They often involve combining accelerometer and magnetometer data for their compensation characteristics, which is a good solution for complementing the drift-free gyroscope [41]. 

A sensor fusion method is often applied to reduce error propagation and obtain the integration process initial conditions [42]. In these cases, the MEMS gyroscope signal is still the basis for orientation estimation, but it is refined with the data from the MEMS accelerometer and magnetic sensors in the Miniature Inertial Measurement Unit (MIMU) [43], where the MEMS gyroscope is not used alone, but together with the MEMS accelerometer and magnetometer.

**Figure 9:**
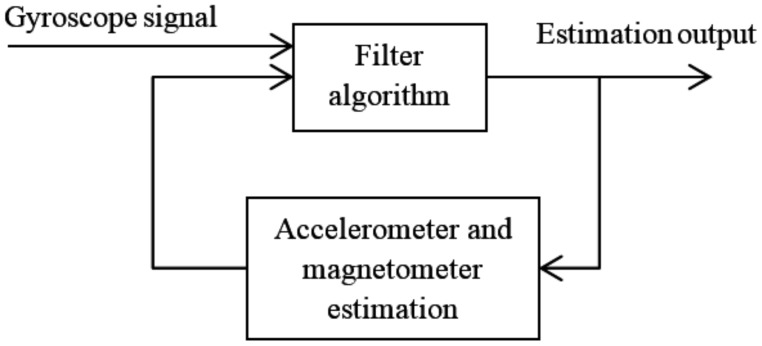
Main structure of the sensor fusion approach using accelerometer and magnetometer data.

The main structure of the sensor fusion approach exploiting accelerometer and magnetometer data is presented in Figure 9. The detailed algorithms depend on different filter algorithms. The most common filter algorithm used is the Kalman filter, which consists of prediction and correction steps and connects for the adaption of accelerometer and magnetometer estimation. 

### 3.3. Simple Filter Algorithms

#### 3.3.1. Low-Pass Filter

A simple digitally implemented RC low-pass filter was applied in the MEMS gyroscope-based motion detection system for noise reduction [25]. The low-pass filter can be described as [44]:

(16) $$y_{i}=y_{i-1}+\alpha (x_{i}-y_{i-1})$$

where *α* is the low-pass cut-off frequency parameter, which has a value from zero to one. Using a suitable parameter value, the high-frequency noise can be filtered out.

#### 3.3.2. High-Pass Filter

Analogous to the low-pass filter that is described above, a simple digital high-pass filter was implemented in a similar way in a MEMS gyroscope-based motion detection system for drift/offset reduction [26]. The digitally implemented high-pass filter can be described as [26]:

(17) $$y_{i}=\beta y_{i-1}+\beta (x_{i}-x_{i-1})$$

where *β* is the parameter of the cut-off frequency for the high-pass filter, which has a value from zero to one; $x_{i}$ and $x_{i-1}$ are the input signals at time points *i* and *i*−1, respectively; and $y_{i}$ and $y_{i-1}$ are the output signals at time points *i* and *i*−1, respectively. 

The high-pass filtering operation was also used to remove most of the sensor measurement biases [45].

#### 3.3.3. Threshold with Delay Method (TWD)

Based on the original threshold method, the threshold with delay (TWD) method is developed with a delay parameter with the aim of not interrupting the continuity of the movement signal when using a threshold method [27]. The algorithm can be described mathematically as:

(18) $$y_{k}=\{\begin{array}{c} x_{k},x_{k}\geq T∥t_{y\leq T}<D\\ 0,x_{k}<T\;\&\;t_{y\leq T}>D \end{array}$$

where *x* and *y* are the input and output signals, *k* is the discrete point in time, and *D* is the delay. With suitable threshold and delay values, the TWD algorithm can be used to filter out the noise around zero and obtain a smooth continuous signal, even if the movements make the signal cross the threshold level several times. The threshold level is usually set to as close to the noise level as possible, but slightly above it [27].

### 3.4. Compensation-Based Algorithms

#### 3.4.1. Drift and Offset Compensator (DOC)

**Figure 10:**
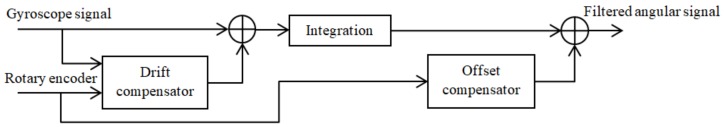
Structure of the DOC.

The drift and offset compensator (DOC) is a model-free method for compensating for the drift and offset in the MEMS gyroscope signals [45]. It employs FIR/IIR filtering techniques and lends itself to implementation in hardware such as DSPs and FPGAs [45]. The simplified main structure of the filtering process of DOC is illustrated in Figure 10, which is derived from Figure 1 in the original paper. 

**Figure 11:**
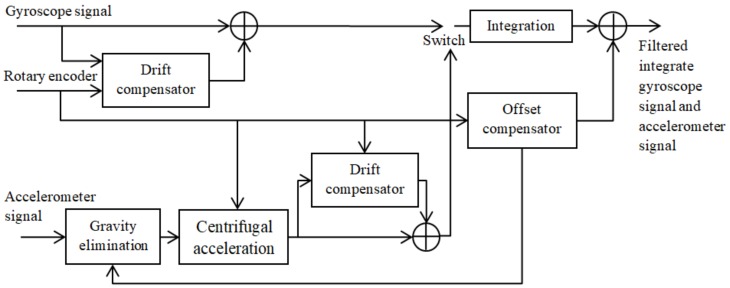
Structure of the enhanced DOC.

To compensate for the drawbacks of the angular rate estimation based on only the encoder signals if the encoder step size is higher than the angle that is passed during the sampling interval or of the same order of magnitude [45], an enhanced DOC is also presented in the paper [45]. As shown in Figure 11, the enhanced DOC employs both an accelerometer and a gyroscope for angular rate estimation.

#### 3.4.2. Compensation Method with Temperature 

This method employs orientation-based gyroscope compensation, including temperature, and further employs a Kalman-based model that uses an orientation sensor and temperature [46]. The corrected value of the gyro sensor is expressed as [46]:

(19) $$G_{Corr}=G_{real}-S_{bias}-t_{bias}-M_{error}-\varepsilon$$

where $G_{real}$ is the gyro sensor value, $S_{bias}$ is the static bias, $t_{bias}$ is the bias due to temperature, $M_{error}$ is the error during motion, and ε is white noise.

The median filter on a moving window of size 7 is used to remove the white noise [46]. Then, static bias prediction is used to capture the static drift parameter for static drift compensation. With a separate chip for measuring the temperature of the sensor, the static sensor data variation with the temperature for each axis is analyzed to determine the temperature bias. The total bias is the sum of the temperature bias and the static bias, which can be expressed as:

*Bias* = *m* × *T* + *C*, (20)

where *T* is the temperature mean, *m* × *T* is the temperature bias, and *C* is the static bias.

After removing the static bias and the bias due to temperature, to eliminate the misalignment error that results in incorrect distribution of the angular velocity along different axes in a tri-axial gyroscope, the Kalman filter is used to remove the noise and compensate for the orientation error by compensating for the value of gyroscope with the values of the orientation sensors [46]. The state space equation is [46]:

(21) $$\theta_{t+\Delta t}=\theta_{t}+\Delta t\times \dot{\theta }$$

where $\theta_{t}$ is the value from the orientation sensor, $\dot{\theta }$ is the value from the gyroscope sensor, and Δt is the sampling time. For the Kalman filter coefficients, the state transition matrix and the observation matrix are $A=\left[\begin{array}{cc} 1 & \Delta t\\ 0 & 1 \end{array}\right]$ and $H=\left[\begin{array}{cc} 1 & 0\\ 0 & 1 \end{array}\right]$, respectively [46]. The processing noise and experiment noise are $Q=\left[\begin{array}{cc} 0.00009 & 0\\ 0 & 0.00009 \end{array}\right]$ and $R=\left[\begin{array}{cc} 0.002 & 0\\ 0 & 0.002 \end{array}\right]$, respectively [46]. The input of the Kalman filter is $X_{input}=\left[\begin{array}{c} Orientation\_angle\\ Gyroscope\_data \end{array}\right]$ [46].

**Figure 12:**
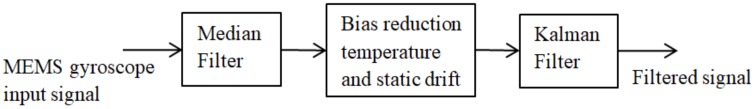
Structure of the compensation method with temperature.

The procedure of the filter method is illustrated in Figure 12. 

This gyro drift compensation method is not implemented in hardware, such as a microprocessor or dsPIC, but over the sensor service layer of the existing Android sensor stack [46].

#### 3.4.3. Compensation Method with Accelerometer and Magnetometer Data

A complementary-filter-based filter is designed for estimating the angle in the application of a mini wearable wireless sensor system for rehabilitation [47]. It has the advantages of a lower computational burden and higher precision than the Kalman filter [47]. However, it requires the accelerometer data and magnetometer data to be analyzed together with the gyroscope data [47]. 

A robust and easy-to-implement method for calibrating an inertial measurement unit (IMU) with an MEMS gyroscope, accelerometer, and often a magnetometer, without any external equipment, is introduced in the paper by D. Tedaldi et al. [48]. The calibration protocol can be summarized as the following steps: Let IMU be static for T seconds. Then, rotate the IMU to a different attitude and wait at least t_wait_ seconds. Finally, repeat the rotation process N times to estimate the parameters by the calibration algorithm. The signal must be averaged over a suitable time interval. The accuracy of the calibration strongly depends on the reliability of the classification between static and motion intervals: static intervals are used to calibrate the accelerometers, while motion intervals are also included between consecutive static intervals for gyroscopes [48].

### 3.5. Category of the Algorithms 

The reviewed articles, with their corresponding algorithms, were categorized into four groups. The details of their classifications, characteristics/functions, supplements/requirements, related applications, and numbers of papers are summarized in Table 1.

In Table 2, these algorithms were also categorized into groups, and details regarding the main functions, advantages, disadvantages, and numbers of studies are specified.

## 4. Discussion

A systematic review was performed with a focus on error reduction algorithms in MEMS gyroscope-based motion analysis systems within the area of human motion analysis or with a clear potential for use in this area (e.g., for robotics). A total of 17 algorithms have been classified into four categories: Kalman-filter-based algorithms, adaptive-based algorithms, simple filter algorithms, and compensation-based algorithms. The most commonly used solution for MEMS gyroscope error reduction within this area is adaptive-based algorithms, followed by Kalman-filter-based algorithms. The majority of them (12 of 17) showed the potential to be simplified to limit the calculation time. All the algorithms can be implemented in real time and used alone or combined with other algorithms or sensors, e.g., accelerometers and/or magnetometers. They are suitable for use in human motion analysis systems, especially for hand, wrist, and head movements.

Kalman-filter-based algorithms are commonly applied for error compensation, position correction, and orientation estimation in different areas. With a focus on error reduction, related Kalman-filter-based algorithms, including the Kalman filter, discrete Kalman filter in an optimal way, simplified basic Kalman filter, and Kalman-filter-based position estimation algorithms, are presented in this paper. They are all based on the Kalman filter and have the main function of noise reduction and offset/drift signal prediction and estimation, including human tremor reduction, drift estimation, and yaw correction during motion analysis.

Adaptive-based and related algorithms, such as LMS, the adaptive sliding mode controller, the adaptive bandpass filter, WFLC, and the sensor fusion algorithm, were used for noise reduction, error compensation and estimation, and human tremor reduction, modeling, and estimation. Sensor fusion algorithms often combine accelerometer and magnetometer data to achieve their compensation characteristics. 

Some simple filter algorithms, such as the low-pass filter, high-pass filter, and TWD algorithm, were also applied to reduce signal errors, e.g., noise, drift/offset, and integration drift. They have simple characteristics and thus simple functions. They are simple and easy to implement in real time, but only with simple functions. They are good solutions for simple tasks. However, a better result is usually achieved if they are combined with other algorithms.

Compensation algorithms include DOC, compensation with temperature, compensation with accelerometer and magnetometer data, and the IMU calibration method. They can be applied for noise reduction and angle estimation by compensating for the related gyroscope errors, e.g., drift/offset and bias due to temperature. The compensation algorithms are satisfactory solutions for complementing the drift-free gyroscope, but require other resources in combination to perform their function, including other filter algorithms and additional hardware, such as accelerometers and magnetometers, or even the same sensor unit as a reference.

## 5. Conclusions

This paper presents a systematic overview of signal processing algorithms with a focus on MEMS gyroscope error reduction for human motion analysis systems. Sixteen MEMS gyroscope-related signal processing papers that were published in the past 10 years were reviewed to evaluate their functions, error reduction/minimization, and signal improvements. All of them are within the area of human motion analysis applications or have a clear potential for use in this area. Seventeen algorithms, which were categorized into four main groups (Kalman-filter-based algorithms, adaptive-based algorithms, simple filter algorithms, and compensation-based algorithms), were investigated and summarized in terms of their characteristics, functions, supplements/requirements, related applications, advantages, and disadvantages. Some signal processing algorithms can be used alone for MEMS gyroscope error reduction, whereas others should be used with other signal processing algorithms to achieve better results. Some algorithms combine MEMS gyroscope data with data from other sensors as accelerometers or both accelerometers and magnetometers, while some require a reference sensor model. This study also showed the possibility of simplifying the algorithms for a limited calculation capacity and real-time implementation. The algorithms that were investigated in this paper are useful for signal error reduction and signal quality improvement for MEMS gyroscope-based human motion analysis. With a focus on MEMS gyroscope-based movement measurement, the paper aims at being a user guide on when to use which algorithms in related studies.

## Figures and Tables

**Table 1:** Reviewed algorithms with references.

Algorithm Group	Algorithms	Characteristics/Functions with References	Number of Papers	Supplements/Requirements				Applications
				Limited Calculation Time	Real Time/on- or off-Line	Working Environment	Need for Combination	
Kalman-filter-based algorithm	Kalman filter (KF)	Noise reduction; signal prediction and estimation: human tremor estimation and modeling [35]; physiological tremor estimation [36]; drift compensation together with a compensation method [47]	3	- ^1^	Real-time estimation of tremor parameters	MATLAB	Together with the WFLC algorithm to estimate the instantaneous tremor frequency; together with a compensation method to compensate for the drift	Tremor motion extraction from voluntary movement (hand motion/wrist rotation) estimation with MEMS gyroscope; Drift compensation for MEMS gyroscope in mobile devices for human motion analysis
	Discrete KF in an optimal way	Optimal estimation of the bias drift and noise from MEMS gyroscopes signals [37]	1	Simplification of KF implementation	Real-time processing	Digital signal processor (DSP)	Without needing other sensor’s information	MEMS gyroscope (not human motion in the article, but with potential to be used in human motion analysis)
	Simplified basic Kalman filter	Noise reduction [25]; temperature drift estimation [26,27]	3	Within limited calculation time	Real time	MATLAB and DSPs	Can be used alone	Gyroscopic head-borne computer mouse
	KF based position estimation algorithm	Yaw correction during position estimation [38]	1	- ^1^	Real time	MATLAB	Need additional accelerometer and magnetometer/compass data	Hand motion and hand position estimation
Adaptive-based algorithm	Least Mean Square (LMS)	Noise reduction [25]; tremor modeling [35]	2	Within limited calculation	Real time	MATLAB and DSPs	Can be used alone	Gyroscopic head-borne computer mouse
	Adaptive slide mode controller	Fabrication imperfection compensation, external disturbances reduction [39]	1	- ^1^	Real time	MATLAB/Simulink	Need a reference model (ideal oscillator)	Oscillatory motion by MEMS z-axis vibrating gyroscope system (with potential to be used in human motion analysis)
	Adaptive bandpass filter	Typical noise/pathological tremor reduction [40]	1	Simple and easy to implement	Real time	MATLAB Simulink	Both gyroscope and accelerometer	Volitional hand movement
	WFLC	Noise reduction [25,27]; human tremor frequency tracking [35]; physiological tremor estimation [36]	4	Within limited calculation time	Real time	MATLAB and DSPs	Can be used alone	Gyroscopic head-borne computer mouse
	BMFLC	Human tremor frequency tracking [35]; physiological tremor estimation [36]	2	- ^1^	Real time	MATLAB	Can be used alone	Tremor motion extraction from voluntary movement (hand motion/wrist rotation) estimation with MEMS gyroscope
	Sensor fusion	Integration drift error reduction and error propagation reduction during orientation/position estimation [42,43]; drift compensation [41];, etc.	3	Developed with shorter computation time (than rotation matrix)	Real time [42]	MATLAB; Mobile phone API, IoT	Need to exploit accelerometer and magnetometer aiding sensors, and need reference data	3D human movement analysis; rehabilitation application, monitoring dynamic changes of movement for clinical prognosis
Simple filter algorithm	Low-pass filter	Noise reduction [25]	1	Within limited calculation time	Real time	MATLAB and DSPs	Can be used alone	Gyroscopic head-borne computer mouse
	High-pass filter	Drift/offset reduction [26]; bias reduction [45]	2	Within limited calculation time	Real time	MATLAB and DSPs	Can be used alone	Gyroscopic head-borne computer mouse
	TWD	Noise reduction around zero within the threshold [27]	1	Within limited calculation time	Real time	MATLAB and DSPs	Followed with other algorithms to obtain better results	Gyroscopic head-borne computer mouse
Compensation-based algorithm	Drift and offset compensator (DOC)	Drift/offset compensation [45]	1	Low computational demands	Real time	DSPs and FPGAs	Based on encoder measurement. Combination of encoder and even MEMS accelerometer	Demanding robotic and mechatronic systems; parallel or serial kinematic machines such as industrial manipulators (with the potential to be used in human motion analysis)
	Compensation method with temperature	Noise reduction and drift compensation (including bias due to temperature) [46]	1	- ^1^	Real time	Android	Combination of Median filter, Kalman filter	Drift compensation for MEMS gyroscope in mobile devices that are in motion, static, or with temperature variance. This method optimally filters drift to be usable in MARG, IMU, indoor navigation and human activity classification
	Compensation method with accelerometer and magnetometer	Noise reduction and angle estimation [47]	1	Required less computation	Real time	Microprocessor	Need to combine with MEMS accelerometer and magnetometer data	Capture real time body movement with a mini wearable wireless sensor system for rehabilitation
	IMU calibration method	Absolute error reduction; calibration parameter estimation [48]	1	Robust and easy to implement	Real time	MATLAB	An IMU consists of a tri-axial MEMS gyroscope, an accelerometer and often a magnetometer. Does not require other external equipment	Low-cost IMU sensors equipped on smartphones and similar devices for motion analysis of robotics. It is possible to use it for human motion analysis

^1^ ‘-’ indicates that no information could be found in the original paper.

**Table 2:** Summary of error reduction algorithms in groups with corresponding functions, advantages, and disadvantages.

	Main/Common Functions	Advantages	Disadvantages	Number of Studies
Kalman-filter-based algorithm	Noise reduction including tremors Signal prediction and estimation Offset/drift error estimation Yaw correction	One of the most common signal processing algorithms for the MEMS gyroscope Can be implemented in real time Can be simplified for limited calculation capacity	Sometimes requires information from another sensor, e.g., MEMS accelerometer or magnetometer or must work together with other algorithms for specific applications, e.g., with WFLC for tremor motion extraction	8
Adaptive-based algorithm	Noise reduction Tremor modeling and estimation Tremor modeling and estimation Drift compensation	The most common signal processing algorithm for the MEMS gyroscope Can be implemented in real time Can be simplified for limited calculation capacity	Some algorithms (e.g., sensor fusion) must be combined with other sensors, e.g., accelerometer or magnetometer Or require a reference model	13
Simple filter algorithm	Noise reduction Or offset/drift reduction, integration drift reduction	Simple to implement Can be implemented in real time	Usually for a single simple function Need to be combined with other algorithms for a better result Some algorithms need to be combined with other sensors, e.g., MEMS accelerometer Common in practical applications but not in research publications	4
Compensation-based algorithm	Offset/drift compensation Noise reduction Angle estimation	Can be implemented in real time Can demand low computation	Usually combined with other hardware, e.g., encoder, accelerometer, or magnetometer Combined with other algorithms, e.g., median filter, Kalman filter The accuracy of the calibration method strongly depends on the reliability of the classification between static and motion intervals	4
